# The positive impact of smoking on poor sleep quality is moderated by IGF1 levels in cerebrospinal fluid: a case-control study among Chinese adults

**DOI:** 10.3389/fpsyt.2024.1392732

**Published:** 2024-05-10

**Authors:** Ligang Shan, Yuyu Wu, Jiaying Lao, Mingwei Ma, Xingguang Luo, Ke Zheng, Weiming Hu, Yimin Kang, Fan Wang, Yanlong Liu, Yali Xu, Xiaoya Jin

**Affiliations:** ^1^ Department of Anesthesiology, The Second Affiliated Hospital of Xiamen Medical College, Xiamen, China; ^2^ School of Mental Health, Wenzhou Medical University, Wenzhou, Zhejiang, China; ^3^ Department of Psychiatry, Yale University School of Medicine, New Haven, CT, United States; ^4^ Zhejiang Provincial Clinical Research Center for Mental Disorders, The Affiliated Wenzhou Kangning Hospital, Wenzhou Medical University, Wenzhou, China; ^5^ Department of Psychiatry, The Third Hospital of Quzhou, Quzhou, China; ^6^ Psychosomatic Medicine Research Division, Inner Mongolia Medical University, Hohhot, China; ^7^ Beijing Huilongguan Hospital, Peking University, Beijing, China; ^8^ Infection Control Department, The First Affiliated Hospital of Wenzhou Medical University, Wenzhou, Zhejiang, China; ^9^ Department of Infectious Diseases, The First Affiliated Hospital of Wenzhou Medical University, Wenzhou, Zhejiang, China

**Keywords:** smoking, PSQI score, sleep disturbances, IGF1, moderation

## Abstract

**Objective:**

Previous research indicates associations between cigarette smoking, insulin-like growth factor-1 (IGF1), and sleep disturbances. This study aimed to examine the association between smoking and sleep quality and investigate the moderating role of IGF1.

**Methods:**

This case-control study involved 146 Chinese adult males (53 active smokers and 93 non-smokers) from September 2014 to January 2016. Sleep quality and disturbances were evaluated using the Pittsburgh Sleep Quality Index (PSQI), which includes seven scales. Pearson correlation analysis and logistic regression analysis were utilized to examine the link between IGF1 levels in cerebrospinal fluid (CSF) and PSQI scores. The effect of IGF1 was assessed using the moderation effect and simple slope analysis, with adjustments made for potential confounders.

**Results:**

Active smokers exhibited significantly higher global PSQI scores and lower IGF1 levels in CSF compared to non-smokers. A significant negative correlation was observed between IGF1 and PSQI scores (â = -0.28, P < 0.001), with a stronger association in non-smokers (Pearson r = -0.30) compared to smokers (Pearson r = -0.01). Smoking was associated with higher global PSQI scores (â = 0.282, P < 0.001), and this association was moderated by IGF1 levels in CSF (â = 0.145, P < 0.05), with a stronger effect at high IGF1 levels (Bsimple = 0.402, p < 0.001) compared to low IGF1 levels (Bsimple = 0.112, p = 0.268). Four subgroup analysis revealed similar results for sleep disturbances (Bsimple = 0.628, P < 0.001), with a marginal moderation effect observed on subjective sleep quality (Bsimple = 0.150, P = 0.070). However, independent associations rather than moderating effects were observed between IGF1 and sleep efficiency and daytime disturbance.

**Conclusion:**

We provided evidence to demonstrate the moderation effect of IGF1 on the relationship between smoking and sleep in CSF among Chinese adult males.

## 1 Introduction

Poor sleep quality has become increasingly prevalent in various population over the past decades, affecting more than a quarter of the population worldwide (1, 2). Existing epidemiological surveys have reported that approximately one-third of adults suffer from one or more sleep disorders during their aggregate lifetime (3). Of note, sleep disorder often coexists with physical health conditions and psychological comorbidities (4, 5), which in turn exacerbate the symptoms that profoundly disturb sleep quality (6). Thus, sleep disorders have gained widespread concern from all walks of life as a major public health issue and a health management challenge.

Multiple factors such as unhealthy lifestyles (smoking, drinking, sedentary, diet), chronic diseases (mental illness, metabolic diseases) housing conditions, and socializing status may contribute to abnormal sleep patterns. Among these, cigarettes smoking has been shown to be detrimental to healthy sleep in several studies. A recently published meta-analysis indicated that smoking carried a higher risk of developing sleep-related issues than non-smoking (7), and sleep disorders similarly increased the difficulties for smoking cessation (8). Nevertheless, it has been reported that sleep disorders were still prevalent among smokers despite their intense quitting attempts (9). Indeed, large amounts of nicotine contained in cigarette smoke can readily penetrate the blood-brain barrier, rapidly distribute throughout the brain (10), and stimulate nicotinic receptors to release a series of neurotransmitters that independently or interactively regulate the sleep-wake cycle, thereby exacerbating sleep disorders and affecting overall sleep quality (11, 12).

Moreover, sleep is associated with the optimal production and secretion of hormones, modulated by neuroendocrine signals (13). Recently, increasing interest has been devoted to exploring neurotrophic factors such as Insulin-like growth factor-1 (IGF1). Based on the previous studies, IGF1 is a hormone that plays a crucial role in the regulation of cell growth, differentiation, and metabolism (14). In population-based studies, high levels of peripheral IGF1 were found to be associated with better sleep quality (15). Epidemiological studies have shown that lower levels of IGF1 have been observed in individuals with chronic insomnia, while individuals with sleep extension have significantly higher levels of IGF1 concentrations in the blood compared to individuals with habitual sleep (15, 16). IGF1 has been shown to have both neuroprotective and neurorestorative effects (17), and several studies have suggested that IGF1 may have a protective effect against the negative effects of smoking on sleep. For example, one study found that chronic nicotine exposure has been found to cause sleep disturbance in rats (18), and IGF1 supplementation can improve sleep quality in rats (19). However, there were inconsistent associations of tobacco exposure with IGF1, as well as differences in IGF1 levels between smokers and non-smokers (20–22). Some studies have not found the differences in the effect of smoking on sleep quality at different levels of IGF1, indicating that the relationship between these factors may be complex and multifaceted. Therefore, the aim of this study was to investigate the effect of IGF1 in cerebrospinal fluid (CSF) on the association between smoking and sleep quality.

## 2 Materials and methods

### 2.1 Study population

Considering the low proportion of female smokers in China (2.7%) (23), males were mainly recruited for the present study. The study design and population have been described in detail previously (24). Briefly, 191 subjects without fatal diseases who were scheduled for anterior cruciate ligament (ACL) reconstruction surgery were enrolled from September 2014 to January 2016 in this study. Information on sociodemographic data (age, marriage, and living) and lifestyles was obtained using interview questionnaire. Clinical information (personal and family history of diseases, history of substance abuse and dependence) was collected based on self-report and confirmed by family members. Physical examination (height and weight) was performed by a trained nurse, and body mass index (BMI) was calculated as weight in kilograms divided by height in meters squared. After excluding those with a family history of psychiatric diseases or systemic or central neurological diseases diagnosed by the Mini International Neuropsychiatric Interview, a total of 146 eligible adult males, comprising active smokers (n = 53) and non-smokers (n = 93), were recruited finally. None of the subjects had a history of alcohol abuse or psychiatric diseases identified by the Diagnostic and Statistical Manual of Mental Disorders (4th Edition). This study was conducted following the Declaration of Helsinki, approved by the Institutional Review Board of Inner Mongolian Medical University, and all the participants provided their written informed consent.

### 2.2 Biosamples collection and laboratory tests

The CSF biosamples were derived from lumbar puncture, the details of which have been elaborated in the previous literature (24). On the morning before ACL reconstruction surgery, a trained and licensed anesthesiologist performed the lumbar puncture operation on the subjects under local anesthesia (using 3 mL of 0.5% ropivacaine), thus collecting 5 mL CSF samples intrathecally. Each sample was distributed into 0.5mL-tubes and immediately stored in a -80°C refrigerator for determination within 24 hours. The entire procedure from hospitalization to surgery took no more than 2 days, during which the subjects were not required to quit smoking.

The levels of IGF1 in CSF were measured using atomic absorption spectrophotometry by professional laboratory technicians. The whole process of detection was in accordance with the principle of double-blind.

### 2.3 Definition of smoking

Non-smokers were defined as subjects who never smoked during their whole life without a history of substance abuse or dependence. Active smokers were those who smoked at least 10 cigarettes a day lasting for over one year. Otherwise, smokers in between–those who smoked less than 10 cigarettes/day–were excluded.

### 2.4 Assessment of PSQI

The Pittsburgh Sleep Quality Index (PSQI) is a recognized comprehensive measurement for subjective self-assessment of sleep quality and disturbances within an interval of the past month that was widely used in clinical practice and research, which can identify good and poor sleepers with high specificity, sensitivity and accuracy (25). Guided by the Chinese version of PSQI (26), all participants had to respond on a four-point Likert scale (from 0 to 3, indicating “no difficulty” to “severe difficulty”). Nineteen individual items were integrated into seven subscales: subjective sleep quality, sleep latency, sleep duration, habitual sleep efficiency, sleep disturbances, use of sleeping medication, and daytime dysfunction (25). The sum of these seven components generated a global score ranging from 0 to 21, with higher scores indicating poorer sleep quality and vice versa. Because fewer participants took sleeping medications, the other six subgroups were included in the subsequent analysis.

### 2.5 Statistical analysis

Categorical variables were described as number (percentage) and compared by Chi-square test. According to the normality distribution (mainly by the Shapiro-Wilk normality test), normally and skewed distributed continuous variables were presented as mean ± standard deviation and median (interquartile range), and the differences between groups were compared using independent t-test and Mann-Whitney *U* test, respectively. The correlation between the IGF1 levels and PSQI scores was examined by Pearson correlation analysis in the active smoking and non-smoking groups. We conducted the traditional linear regression model to investigate the interactive effect of IGF1 and cigarette dependence on PSQI score. Subsequently, a multivariate logistic regression model was employed to elucidate the associations between IGF1 levels in CSF and six subgroups of PSQI in all the subjects. Both linear regression and logistic regression models were adjusted for age (years, continuous), BMI (kg/m^2^, continuous), marriage (married/unmarried), and living (living alone/living with one roommate/living with family members). Furthermore, a moderation effect analysis and simple slope analysis were applied to assess the moderating effect of IGF1 on the relationship between smoking and PSQI scores and the significant components of PSQI. All statistical analyses were conducted using the R software (version 4.2.0, R Foundation for Statistical Computing). The moderation analysis was performed using the “Bruce R” package. All tests were two-tailed and a *P* < 0.05 was considered statistically significant.

## 3 Results

### 3.1 Basic characteristics of study population

The basic characteristics of the 146 participants are presented in **Table 1**, with a smoking rate of 36.3%. As demonstrated in **Table 1**, active smokers were more likely to be older, unmarried, living alone, and have a higher BMI (all *P* < 0.05). Compared with non-smokers, the levels of IGF1 in CSF were significantly lower among active smokers (median levels of 33.0 ng/mL vs. 35.1 ng/mL, *P* < 0.001), while significantly higher in PSQI scores were observed (4.02 ± 2.27 vs. 2.60 ± 2.46, *P* < 0.001), particularly for sleep disturbance, sleep latency, and sleep quality. In addition, no differences were found for blood pressure and other components of PSQI between the two groups (*P* > 0.05).

**Table 1:** Comparisons of descriptive characteristics between non-smokers and active smokers.

Variable	Non-smokers (n = 93)	Active smokers (n = 53)	*P*
Age, y	27 (22, 35)	31 (25, 35)	0.013*
BMI, kg/m^2^	24.2 (22.1, 26.7)	25.4 (23.9, 27.4)	0.035*
SBP, mmHg	130 ± 13	128 ± 13	0.200
DBP, mmHg	75 ± 9	77 ± 12	0.300
IGF1, ng/mL	35.1 (32.3, 36.9)	33.0 (30.3, 35.9)	0.029*
PSQI Component Scores	2.60 ± 2.46	4.02 ± 2.27	<0.001
Sleep Quality	0.51 ± 0.64	0.72 ± 0.60	0.028
Sleep Latency	0.32 ± 0.61	0.72 ± 0.63	<0.001
Sleep Duration	0.66 ± 0.71	0.66 ± 0.76	>0.900
Sleep Efficiency	0.08 ± 0.27	0.21 ± 0.63	0.400
Sleep Disturbance	0.40 ± 0.57	0.87 ± 0.48	<0.001
Sleep Medication	0.05 ± 0.27	0.02 ± 0.14	0.400
Daytime Dysfunction	0.59 ± 0.76	0.83 ± 0.91	0.140
Marriage, n (%)			0.011
Married	50 (53.8)	17 (32.1)	
Unmarried	43 (46.2)	36 (67.9)	
Living, n (%) *^a^*			0.003**
Living alone	6 (6.4)	7 (13.2)	
Living with 1 roommate	25 (26.9)	3 (5.7)	
Living with family members	62 (66.7)	43 (81.1)	

BMI, body mass index; SBP, systolic blood pressure; DBP, diastolic blood pressure; IGF1, insulin-like growth factor-1; PSQI, Pittsburgh Sleep Quality Index.

Marriage and living status were presented as number (%) and other data were presented as mean ± standard deviation or median (interquartile range) according to the normality distribution. P values between non-smokers and active smokers were calculated using Chi-square test for categorical variables and independent t-test or Mann-Whitney U test for continuous variables.

^a^The difference of living status was compared using Fisher’s exact test.

*P < 0.05, **P < 0.01.

### 3.2 Correlation between IGF1 levels in CSF and PSQI scores and fractional latitude score in different groups

**Figure 1:**
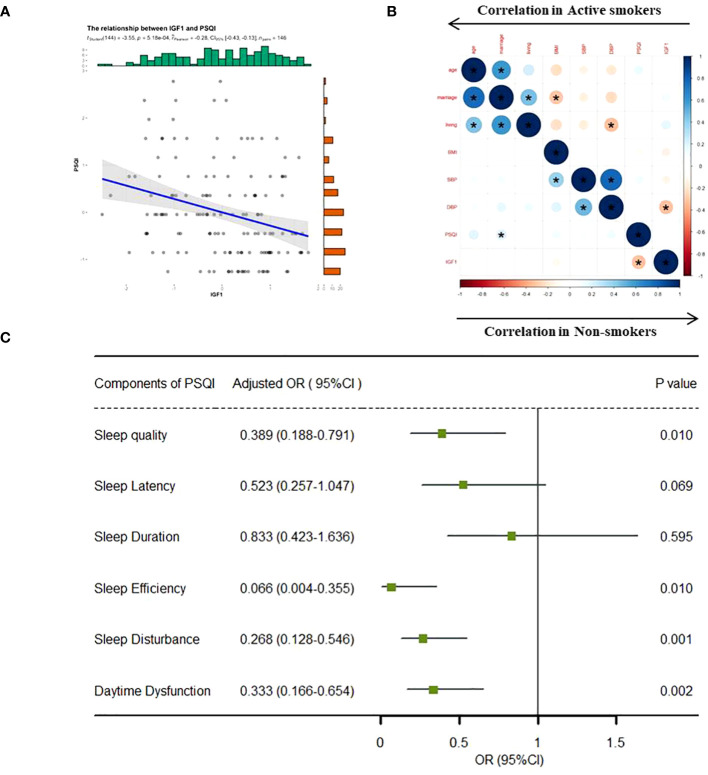
Correlation analysis between IGF1 and PSQI scores. IGF1, insulin-like growth factor-1; PSQI, Pittsburgh Sleep Quality Index; OR, odds ratio; CI, confidence interval. **(A)** Linear regression model was used to analyze the relationship between IGF1 levels and PSQI scores in all the groups. **(B)** Bivariate correlation matrix for the study variables in non-smokers and active smokers using pearson correlation analysis. *P < 0.05. **(C)** IGF1 (divided into two groups by the median level) and seven components of PSQI (yes/no) were included into the logistics regression model as dichotomous variables to assess the associations between the two. The linear regression and logistic regression models were adjusted for age, body mass index, marriage and living. Statistical significance (*P* < 0.05) was denoted in boldface.

In the total population, the levels of IGF1 in CSF were negatively correlated with PSQI scores (β = -0.28, *P* < 0.001) (**Figure 1A**). However, the correlations differed across groups, where a significant negative correlation existed in non-smokers (Pearson r = -0.30, *P* < 0.001) but no correlation in active smokers Pearson r = -0.01, *P* = 0.703) (**Figure 1B**). Furthermore, the relationships between IGF1 levels (divided into two groups by the median) and six subgroups of PSQI were assessed by logistic regression model (**Figure 1C**). After adjusting for age, BMI, marriage and living, the levels IGF1 in CSF were inversely associated with sleep disturbances (OR = 0.268, 95% CI = 0.128-0.546, *P* = 0.001), as well as sleep quality, habitual efficiency, and daytime dysfunction (all *P* < 0.05).

### 3.3 Moderation effect of IGF1

**Figure 2:**
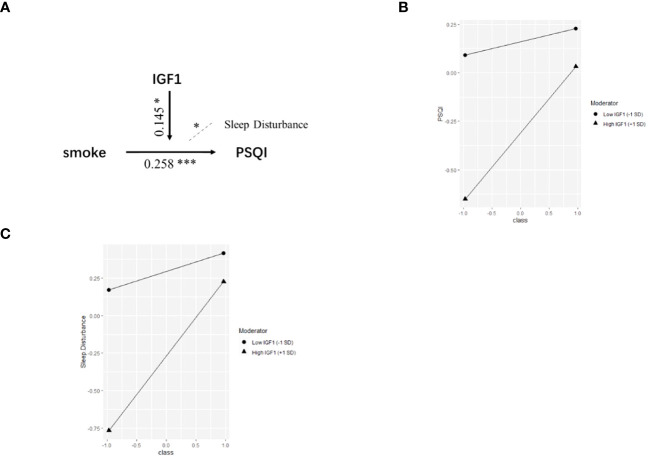
The moderation by IGF1 for the association of smoking with PSQI. IGF1, insulin-like growth factor-1; PSQI, Pittsburgh Sleep Quality Index. **(A)** Conceptual model of moderation analysis regarding IGF1 as moderator. **(B, C)** Simple slope analysis for the moderation effect of IGF1 on the relationship between smoking and PSQI scores **(B)** as well as sleep disturbance **(C)**. Class (-1) referred to non-smokers and Class (+1) referred to active smokers. The two lines represented the regression line of the association of smoking with PSQI scores **(B)** and sleep disturbance **(C)** when IGF1 was at low (circle) or high (triangle) levels. All data was included in the model as numerical variables and reported as moderation analysis. All the models were adjusted for age, BMI, marriage, and living. *P < 0.05, ***P < 0.001.

**Figure 3:**
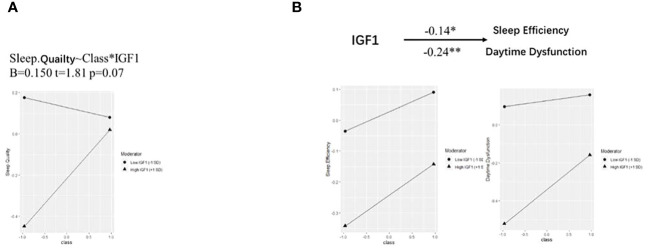
Simple slope analysis for the moderation effect of IGF-1 on PSQI components. IGF-1, insulin-like growth factor-1. **(A, B)** Simple slope analysis for the moderation effect of IGF-1 on the relationship between smoking and sleep quality **(A)** as well as sleep efficiency and daytime dysfunction **(B)**. Class (-1) referred to non-smokers and Class (+1) referred to active smokers. The two lines represented the regression line of the association of smoking with PSQI components when IGF-1 was at low (circle) or high (triangle) levels. All data was included in the model as numerical variables and reported as moderation analysis. All the models were adjusted for age, BMI, marriage, and living. *P < 0.05, **P < 0.01.

Based on the correlations in **Figure 1**, we further estimated the moderating effect of IGF1 on the relationship between smoking and PSQI scores using moderation analysis (**Table 2**). The first step of moderation analysis is to assess the association between independent and outcome variables. As shown in Model 1 (**Table 2**), smoking was positively associated with PSQI scores after adjusting for potential confounders (β = 0.282, 95% CI = 0.135-0.428, R^2 = ^0.109, *P* < 0.001). In the second step, moderating variables were introduced by including the interaction terms in the linear model. According to Model 3 (**Table 2**), the positive impact of smoking on PSQI scores was moderated by IGF1 levels in CSF (β = 0.145, 95% CI = 0.004-0.285, R2 = 0.155, P < 0.001) (**Figure 2A**), with R2 increasing from 0.109 in Model 1 to 0.155 in model 3 and with F values increasing from 5.325 in Model 1 to 5.667 in Model 3 (ΔF = 0.342, p<0.05) (**Table 2**). To elucidate the moderating role of IGF1 more clearly, we grouped all the subjects by the median level of IGF1 and performed simple slope analysis to investigate the impact of smoking on PSQI scores at different levels of IGF1 in CSF (**Figure 2B**). The positive predictive effect of smoking on PSQI scores was significantly increased in individuals with higher level of IGF1 (Bsimple = 0.402, *P* < 0.001), while it was weakened in those with a lower level of IGF1 (Bsimple = 0.112, P = 0.268). Moreover, we also assessed the moderating effect of IGF1 in four subgroups of PSQI which were significant in the logistic model (**Figure 1C**). Similar results were obtained in sleep disturbances (Bsimple of 0.628 and 0.200 in the groups with high and low IGF1 levels, *P* < 0.001, **Figure 2C**), while a marginal moderating effect of IGF1 on sleep quality (Bsimple = 0.150, *P* = 0.070, **Figure 3A**). However, there were independently negative associations rather than moderation between IGF1 and habitual efficiency (β = -0.14, *P* < 0.05) and daytime dysfunction (β = -0.24, *P* < 0.01, **Figure 3B**).

**Table 2:** Linear regression analysis for the moderation effect of IGF1 on the relationship between smoking and PSQI scores.

	Model 1		Model 2		Model 3	
	β	t	β	t	β	t
Age	0.121	1.233	0.167	1.440	0.122	1.275
BMI	0.104	1.451	0.073	0.904	0.073	1.035
Marriage	-0.006	-0.058	0.026	0.198	-0.025	-0.234
Living	0.034	0.400	0.031	0.322	0.046	0.555
Smoke	0.282***	3.794	–	–	0.258***	3.525
IGF1	–	–	-0.268**	-3.334	-0.212**	-2.967
Smoke × IGF1	–	–	–	–	0.145*	2.042
R²	0.109***		0.093***		0.155***	
F	5.325 (5,140)		3.979(5,140)		5.667 (7,138)	

IGF1, insulin-like growth factor-1; PSQI, Pittsburgh Sleep Quality Index; BMI, body mass index. Model 1 was the linear regression model including smoke as the independent variable and PSQI as the dependent variable. Model 2 was the linear regression model including IGF1 as the independent variable and PSQI as the dependent variable. Model 3 included both IGF1 and smoke × IGF1 terms as independent variables based on Model 1. All the models were adjusted for age, BMI, marriage, and living. All data were reported as moderation analysis. *P < 0.05, **P < 0.01,***P < 0.001.

## 4 Discussion

In the present study, we found global PSQI scores were significantly higher, while IGF1 levels in CSF were lower in active smokers than non-smokers. In addition, there was a significant negative correlation between CSF IGF1 level and global PSQI score, especially in non-smokers. Furthermore, smoking was positively associated with global PSQI scores (β = 0.282, *P* < 0.001), which was moderated by IGF1 levels in CSF (β = 0.145, *P* < 0.05). Our study revealed that IGF1 played a moderating role in the process of smoking-induced sleep disorders, which, to some extent, could provide new insights into the association between cigarette smoking and sleep disorders.

Cigarette smoking is one of the major known contributors to sleep disorders. It has been reported that the smoking rate among poorer sleepers is significantly higher than that of the general population (9). A study dating back to the 1990s showed that smoking was significantly positively associated with sleep disorders (27), which was subsequently supported by various studies in different populations from different regions (28, 29). Sleep quality varies with the characteristics and intensity of smoking, a conclusion further supported by the present study. In addition. there are studies that support the possible interactions between smoking, sleep quality, and respiratory problems. For example, a study by Jang et al. found that smoking was associated with an increased risk of obstructive sleep apnea (OSA), a common respiratory disorder that can lead to poor sleep quality (30). Another study by Caliri et al. found that smoking was associated with increased inflammation and oxidative stress in the airways, which could contribute to the development of respiratory problems such as chronic obstructive pulmonary disease (COPD) (31). Moreover, some studies found that smoking was associated with poor sleep quality, and that the combination of smoking and poor sleep quality was associated with increased inflammation and oxidative stress, suggesting that these factors may interact to exacerbate respiratory problems (32–34). Overall, the existing literature suggests that there are complex interactions between smoking, sleep quality, and respiratory problems, and that these factors may influence each other in important ways. Further research is needed to fully understand these interactions and their clinical implications.

In the present study, IGF1 levels in CSF were lower in active smokers than non-smokers. Previous studies have shown that circulating IGF1 has the ability to reach the central nervous system through either the blood-CSF barrier or the blood-brain barrier (35, 36). Thus, the lack of an increase in peripheral IGF1 can lead to a deficiency of IGF1 in the brain (36). Smoking has been shown to reduce the level of peripheral IGF1 (20, 37) and destroy the blood-brain barrier (38, 39), which can further explain our results.

The main finding of present study is that there was a significant negative correlation between the IGF1 level in CSF and PSQI score, especially in non-smokers. A low serum IGF1 level has been reported to be associated with sleep-related disease, and longer slow wave time could be associated with increased IGF1 levels (40, 41). A recent case-control study in China indicated that serum IGF1 concentration was negatively associated with chronic insomnia, sleep disorders and anxiety scores (15). Behavioral symptoms of circadian rhythm imbalance and sleep-wake disorders were noted to be improved by increasing or releasing free IGF1 in serum (16, 42). Similarly, animal experiments and epidemiological studies have revealed that sleep disorders might inhibit the IGF1 axis, with circulating IGF1 levels significantly declining after sustained sleep deprivation (43, 44). The underlying mechanisms are complex. IGF1 is known for its neuroprotective properties, activating IGF1 receptor to initiate downstream phosphorylation cascades that regulate transcription, synaptic maturation, inhibits apoptosis, and promote growth, differentiation and metabolism of neuronal cells (35). Firstly, this relationship may be attributed to BDNF/IGF1 regulated neuronal plasticity changes, hypothesized to increase slow wave sleep activity (45, 46). Moreover, IGF1 could facilitate the repair of neurons from hypoxia and improve sleep regulation (47). These studies suggest that IGF1 could improve sleep quality to some extent, which is similar to our results.

Moreover, our results imply that the level of IGF1 might differently influence the relationship between smoking and sleep quality. As mentioned, a high IGF1 level is associated with low PSQI scores in both non-smokers and all participants, indicating that IGF1, like cigarettes, leads to a direct effect on sleep quality. However, for participants with different CSF IGF1 levels, we not only found the independent effects from the two factors (β = 0.258***, t = 3.525 for smoking; β = -0.212**, t = -2.967 for IGF1). In addition, there was a complex interaction (β = 0.145*, t = 2.042). For participants with a low CSF IGF1 level, smoking did not activate sleep problems (β = 0.112, P = 0.268), but in those who with a high CSF IGF1 level, the sleep damage caused by smoking was greatly increased (β = 0.402, P < 0.001).

This finding is intriguing. Elevated IGF1 levels have the ability to regulate sleep, as mentioned above. Numerous studies have shown a positive correlation between IGF1 levels and sleep quality. Therefore, given the independent effects of smoking and IGF1, it is expected that elevated IGF1 levels will counteract the sleep disturbances induced by smoking. In the interaction model and its sub-dimensions, this effect is evident. Specifically, IGF1 exerts a significant protective effect on four dimensions of sleep disturbance: sleep quality, sleep efficiency, sleep disturbance and daytime dysfunction (sleep quality: OR = 0.389, p = 0.010; sleep efficiency: OR = 0.066, p = 0.010; daytime dysfunction: OR = 0.333, p = 0.002; sleep disturbance: OR = 0.268, p = 0.001). It is also noteworthy that an interaction similar to the PSQI results was only observed in the sleep disturbance dimension. Smoking and IGF1 had independent effects on sleep (see **Figure 3**) in the remaining dimensions.

It is likely that this effect is due to the activity of the orexin neurons. A study in 2020 clearly demonstrated that IGF1 in the central nervous system can directly influence the sleep-wake cycle of mice through the activation of orexin neurons. Orexin neurons significantly prolonged sleep duration in mice lacking IGF1 receptors, suggesting the involvement of IGF1 in wakefulness and maintenance via orexin neurons (48). In addition, previous studies have consistently shown a strong link between smoking and orexin expression. Exposure to smoke significantly increases orexin levels, thereby promoting wakefulness (49–51). Consequently, smokers with elevated levels of cerebrospinal fluid IGF1 may experience increased nicotine-induced stimulation of active orexin neurons, leading to this significant positive interaction.

To our knowledge, this is the first study to assess the role of IGF1 in CSF on smoking-induced sleep disorders (indicated by PSQI) in Chinese males. The effect of smoking on PSQI is moderated by different levels of IGF1 in CSF. Admittedly, there are several limitations in this study. First, causal inferences cannot be drawn from the case-control design, and a small sample size may restrict the statistical power to examine associations and moderations. Hence, evidence from prospective studies with larger sample size is warranted. Second, retrospective recall biases may occur using subjective sleep measurements and smoking assessments. Third, anterior cruciate ligament reconstructive surgery may be a potential confounder affecting smoking, sleep quality and biomarkers. Moreover, other potential confounding factor such as obstructive sleep apnea may affect our understanding of the relationship between smoking and sleep. Finally, only men were recruited due to the low smoking rate in women, resulting in limited applicability and generalizability.

## 5 Conclusion

The positive effect of smoking on PSQI scores and sleep disturbances were negatively moderated by the levels of IGF1 in cerebrospinal fluid in Chinese adult males. The results of this study have important clinical implications. Firstly, they highlight the importance of considering IGF1 levels in cerebrospinal fluid when assessing the relationship between smoking and sleep quality. Clinicians may need to monitor IGF1 levels in smokers who report poor sleep quality and consider interventions aimed at increasing IGF1 levels, such as exercise or nutritional supplements. Secondly, the findings suggest that targeting IGF1 may be a potential therapeutic strategy for improving sleep quality in smokers. Future studies are needed to explore the underlying mechanisms and to develop effective interventions. Overall, this study contributes to our understanding of the complex interplay between smoking, IGF1, and sleep quality. The findings have important implications for the development of targeted interventions to improve sleep quality in smokers and for the prevention of smoking-related sleep disturbances.

## Data availability statement

The raw data supporting the conclusions of this article will be made available by the authors, without undue reservation.

## Ethics statement

The studies involving humans were approved by Institutional Review Board of Inner Mongolian Medical University. The studies were conducted in accordance with the local legislation and institutional requirements. The participants provided their written informed consent to participate in this study.

## Author contributions

LS: Writing – original draft. YW: Writing – original draft, Formal Analysis. JL: Writing – review & editing, Resources. MM: Writing – review & editing, Resources. XL: Writing – review & editing. KZ: Writing – original draft, Resources. WH: Writing – review & editing. YK: Writing – review & editing, Resources. FW: Writing – review & editing, Resources. YL: Writing – review & editing, Supervision. YX: Writing – review & editing, Supervision. XJ: Writing – review & editing, Supervision.
